# Supplementary material for: Leveraging transformers and explainable AI for Alzheimer’s disease interpretability
Source: PLoS One. 2025 May 23;20(5):e0322607. doi: 10.1371/journal.pone.0322607 (PMC12101688; doi:10.1371/journal.pone.0322607)
Supplement: Attachment — Submitted filename: Response to reviewers.pdf [file pone.0322607.s003.pdf]

The authors of this manuscript present an interesting approach for the interpretability of Alzheimer’s Disease. While the methodology and concept are effective, I have a few suggestions on various sections of the paper that may require improvements to meet the journal's standards.

1. First, I recommend that the authors consider structuring the introduction into several segments based on relevance. While the introduction begins well, the connections between sentences become weaker as it progresses. Additionally, there are insufficient citations for various claims presented in this section. The overall writing quality of the introduction could be improved. For example, the statement, 'However, there is a gap in incorporating XAI techniques into transformer-based models,' is made but is not followed by an explanation or discussion of the gaps, nor is there any citation provided.
2. Similar improvements are needed in the literature review. While the content is generally acceptable, the writing style should be enhanced. It would be beneficial to ensure relevance between sentences and to review the word choices throughout. For instance, if you spell out 'Convolutional Neural Network' once, you can simply use the abbreviation 'CNN' in subsequent references.
3. The results section requires additional validation considering the claims made by the authors. Currently, the authors only performed a comparative analysis with DeepLabV2. I suggest including a few more off-the-shelf network architectures to better validate your results.
4. Quality of Figure 6 and 7 can be improved.
5. Check all the section heading for inaccurate writing format.
