# Supplementary material for: Leveraging transformers and explainable AI for Alzheimer’s disease interpretability
Source: PLoS One. 2025 May 23;20(5):e0322607. doi: 10.1371/journal.pone.0322607 (PMC12101688; doi:10.1371/journal.pone.0322607)
Supplement: Attachment — Submitted filename: PONE-D-24-39865_reviewer-comments.docx [file pone.0322607.s002.docx]

**Review of Paper PONE-D-24-39865**

**Title:**

**Leveraging Transformers and Explainable AI for Alzheimer’s Disease Interpretability**

**Summary:**

This study addresses Alzheimer’s disease (AD), a progressive brain condition marked by memory loss, cognitive decline, and behavioral changes, affecting one in nine adults over 65. While computer vision applications in MRI-based AD detection have shown accuracies between 80-90%, this study applies advanced transformer models and computer vision to achieve a 99% accuracy rate in categorizing AD stages. By incorporating RNA data and MRI images in near-real-time, this approach improves diagnostic accuracy. The Local Interpretable Model-agnostic Explanations (LIME) method enhances the model's reliability, highlighting essential RNA features and MRI regions for diagnosing AD, thereby making the model’s decisions more interpretable and acceptable for human use.

**Strengths And Weaknesses:**

## Strengths

- It has a thorough explanation of the main idea.
- Clearly explain the methodology.

## Weaknesses

Overall, the writing can be significantly improved to address the following concerns.

- **Introduction:** You can add more references in the related work to show that you check new papers.
- **Results:** The results look good.
