# Supplementary material for: Leveraging transformers and explainable AI for Alzheimer’s disease interpretability
Source: PLoS One. 2025 May 23;20(5):e0322607. doi: 10.1371/journal.pone.0322607 (PMC12101688; doi:10.1371/journal.pone.0322607)
Supplement: Attachment — Submitted filename: Response to Reviewers.docx [file pone.0322607.s005.docx]

**Leveraging Transformers and Explainable AI for Alzheimer’s Disease Interpretability**

Responses and Actions to Reviewer Comments

Dear Editor and Reviewers,

The authors would like to thank the reviewers for providing insightful comments that helped to make the journal paper better. All review judgments and comments are looked at by the authors. Following the reviewers' recommendations, they also offer remarks, feedback, and necessary changes to the work. The authors express their gratitude to all of the esteemed reviewers for their hard work.

Best Regards,

Authors, Manuscript Number: PONE-D-24-39865

**============== Review Comments and Corresponding Actions ======================**

**Additional Editor Comments:**

**Comment-1:**

There are serious deficiencies in the evaluation metrics required for the evaluation of the classification results in the study. Please obtain the missing Receiver Operating Characteristic (ROC) Curve, the area under the ROC curve (AUC) scores, and Cohen's Kappa and Matthews correlation coefficient (MCC) scores completely.

**Response-1:**

Thank you very much for your comments.

We add the Cohen's Kappa, Matthews correlation coefficient (MCC) scores, and AUC values of the experimental models in Table 5. AUCs of all classes are generated from the Receiver Operating Characteristic (ROC) curves in Figure 4 using a vision transformer.

**The following lines are included in Section 5.1, line 352:**

“Table 6 outlines the outcomes obtained by employing a vision transformer across various classes in the testing dataset. The NonDemented class attains the highest accuracy at 99.7%, while the MildDemented class registers the lowest accuracy at 97.31%. The vision transformer accurately classifies 98.6% of cases, with only a few instances of mis-prediction. The accuracy of the MildDemented class is less due to the presence of fewer training images in the dataset. The model performs a little less when there is a small number of training data. Other classes achieve appreciable results. Consequently, the overall accuracy of the model stands at 98.6% for all testing images, with a 95% confidence interval (CI) of [97.41%, 99.36%], indicating a high degree of reliability in classification performance. We derive the CI using 5-fold cross-validation, where the dataset was split into five subsets, training and testing the model iteratively to ensure stable performance across folds. The narrow CI range confirms robustness, as it reflects consistent accuracy with minimal variation across these folds. The CI provides a lower and upper range because it accounts for statistical uncertainty in the sample data, estimating the plausible bounds of the true accuracy. The lower bound (97.41%) represents the minimum expected performance with 95% confidence, while the upper bound (99.36%) suggests the potential peak accuracy under optimal conditions. The corresponding loss and accuracy curves are depicted in Fig. 3 and Fig. 2. The accuracy curves for both training and testing exhibit an initial surge to 80% within the initial 5 epochs, eventually surpassing 95% after 15 epochs. Both curves demonstrate similar trends in loss calculations, reaching saturation after 15 epochs at the same point. Also, the ROC curve is displayed in Fig. 4. The Cohen Kappa coefficient value we obtain is 0.97, indicating almost perfect agreement between the true and predicted labels. Similarly, the Matthews Correlation Coefficient (MCC) is 0.98, reflecting a strong correlation and robust performance of the model across all classes. In Fig. 5, LIME explanations are presented for all four classes of test images. The LIME explanation highlights the specific regions of the images that contributed to the prediction of these classes. Red patches in the explanation images represent areas that positively influenced the model’s accurate prediction. Positive influence implies that these regions were instrumental in the model correctly identifying the class or label. In essence, the presence of red zones indicates that the characteristics or patterns in those areas align with the expected class. In Fig. 6, overlays of the explanations are displayed. The yellow lines point out distinct pixels that played significant roles in predicting the class of these particular images.”

**Comment-2:**

It is recommended to analyze several state-of-the-art models to compare the results in more depth and to make the proposed approach more prominent.

**Response-2:**

Two new state-of-the-art models, including ResNet 50, and EfficientNet Models, are experimented with, and their results are presented in Table 5.

**The following lines are included in Section 5.2, line 406:**

“Additionally, EfficientNet-B0 and ResNet-50 are included for benchmarking, achieving accuracies of 91.57% and 86.59%, respectively, further validating the robustness of our approach. These results collectively highlight the trade-offs between accuracy, computational efficiency, and deployment feasibility. Based on our findings, it can be conclusively stated that the ViT proves to be a highly effective and efficient algorithm for the classification of MRI images related to Alzheimer’s Disease. Table 5 displays the Accuracy, F1-score, Cohen’s Kappa, and Matthew’s correlation coefficient of each model on the dataset. Based on our findings, it can be conclusively stated that the Vision Transformer proves to be a highly effective and efficient algorithm for the classification of MRI images related to Alzheimer’s Disease. Also, Fig. 4 represents the ROC curve of ViT where we can see that ViT achieves high Area Under the Curve (AUC) scores across all Alzheimer’s Disease classes, reflecting its strong ability to distinguish between positive and negative cases for each category. The AUC values 0.98 for ModerateDemented, 0.96 for MildDemented, 1.00 for NonDemented, and 0.99 for VeryMildDemented indicate near-perfect discrimination, with NonDemented reaching an ideal 1.00, aligning with its top accuracy of 99.70%. These results underscore ViT’s robustness in handling class-specific patterns in brain imaging data, even for challenging cases like MildDemented as the number of training images are very low, where accuracy dips slightly and so does the AUC. Also, the Cohen’s Kappa and Matthew’s correlation coefficients are consistent with these findings.”

**Comment-3:**

The literature review table needs to be detailed. Here, more new studies related to the current literature should be included, and new columns such as "data preprocessing/augmentation, originality, plus and minus aspects" should be added. After this, the difference of this study from the literature and its main contribution to the literature should be expressed in more depth by relating it to the studies in the literature.

**Response-3:**

Three new related studies are added accordingly, and the literature review table is split into two separate tables to cover all these aspects mentioned (originality, plus and minus aspects).

**In the Reference section, the following three references are added:**

30. Guven M. Detection of Alzheimer’s and Parkinson’s Diseases Using Deep Learning-Based Various Transformers Models. Eng. Proc. 2024;73(1):4. Presented at the 4th International Electronic Conference on Biosensors, 20–22 May 2024. 629 Available online: https://sciforum.net/event/IECB2024. https://doi.org/10.3390/engproc2024073004.

31. Liu M, Li F, Yan H, Wang K, Ma Y, Shen L, A deep learning system for differential diagnosis of Alzheimer’s disease and mild cognitive impairment using structural MRI. NeuroImage. 2021;225:117496. https://doi.org/10.1016/j.neuroimage.2020.117496.

32. Wang H, Shen Y, Wang S, Xiao T, Deng L, Wang X, Multimodal deep learning for Alzheimer’s disease dementia assessment. Nature Communications. 2020;11(1):1–9. <https://doi.org/10.1038/s41467-020-18187-0>.

**In addition, the following lines are added in Section 2 Literature Review line 116:**

“Wang et al. [32] developed a multimodal deep learning framework for Alzheimer’s Disease dementia assessment, integrating data from neuroimaging, genetic markers, and cognitive tests. Their approach combines CNNs and recurrent neural networks (RNNs) to capture both spatial and temporal patterns in the data. The study shows that multimodal data fusion significantly improves diagnostic accuracy compared to single-modality approaches. The authors reported an accuracy of 92.1% for Alzheimer’s Disease (AD) diagnosis using their multimodal deep learning framework, which integrated neuroimaging, genetic markers, and cognitive tests. This multi-modal approach was quite a new addition to the field of AD diagnosis at the time of publication, but new state-of-the-art algorithms like models can be used for further accuracy improvements. Another study conducted by Liu M et al. [31] proposed a deep learning system for the differential diagnosis of Alzheimer’s Disease (AD) and mild cognitive impairment (MCI) using structural MRI. Their model leverages 3D convolutional neural networks (CNNs) to extract features from brain scans, achieving high accuracy in distinguishing between AD, MCI, and healthy controls. The study highlights the potential of deep learning in automating AD diagnosis and emphasizes the importance of structural MRI as a key biomarker for early detection. reported an accuracy of 88.6% for differentiating Alzheimer’s Disease (AD) from healthy controls and 76.5% for distinguishing mild cognitive impairment (MCI) from healthy controls using their deep learning system based on structural MRI. The accuracy remains relatively low due to the use of CNN-based architectures, and the usability of the moderately accurate model can cause a serious discrepancy in the field of AD detection.”

**The following lines are added in Section 2 Literature Review line 151:**

“Recent work by [30] applied transformer-based models, including Swin Transformer, Vision Transformer (ViT), and Bidirectional Encoder Representation from Image Transformers (BEiT), to classify Alzheimer’s and Parkinson’s diseases using brain imaging data. The study utilized a balanced dataset of 450 brain images, achieving classification accuracy exceeding 80%, with ViT demonstrating the highest performance (94.4% accuracy, 94.7% precision). While the results highlight the efficacy of transformer architectures in disease detection, the study has notable shortcomings. The dataset size (450 images) is relatively small, which may limit the generalizability of the findings. Also, the accuracy is not satisfactory in terms of AD disease diagnosis.”

**Table 1 is partitioned into two parts, Table 1 and Table 2, to add suggested new columns such as "data preprocessing/augmentation, originality, plus and minus aspects."**

**Comment-4:**

Detail the information regarding the dataset along with the justifications in terms of training, validation, test distribution percentages and amounts, and data preprocessing/augmentation.

**Response-4:**

We add detail information on the dataset along with the justifications in terms of training, validation, test distribution percentages, and amounts. **The following part is added to the paper in the 3.1 section, page 7:**

“We utilized the Alzheimer’s dataset from Kaggle [21], which includes gray MRI scans of the brain from individuals in various stages of Alzheimer’s disease. The dataset encompasses four classes: MildDemented, ModerateDemented, NonDemented, and VeryMildDemented. It is organized into two folders: Train and Test, comprising a total of 5121 training images across the four classes and 1379 test images. **Table 3 illustrates the distribution of images in the dataset.** This dataset is valuable for predicting Alzheimer’s disease stages using computer vision algorithms. Given the limited size of the test set, we allocated 10% of the training dataset for validation to ensure a robust evaluation of the model’s performance. This approach was necessary because the dataset did not provide a separate validation set, and splitting the training set further would have risked overfitting due to the imbalanced class distribution. Using a small portion of the test set for validation allowed us to tune hyper-parameters and monitor model performance during training without significantly compromising the final evaluation on the remaining test data.”

**Reviewer#3 Comments:**

**Comment-1:**

The manuscript presents a valuable contribution to Alzheimer’s disease detection using advanced transformers and computer vision techniques. The study is well structured and demonstrates impressive accuracy. To further enhance the work, we recommend citing "Detection of Alzheimer’s and Parkinson’s Diseases Using Deep Learning-Based Various Transformers Models" (<https://doi.org/10.3390/engproc2024073004>) to provide additional context and strengthen the research foundation. Overall, we suggest minor revisions and look forward to the updated submission.

**Response-1:**

**We appreciate your constructive suggestion. Thank you.**

The paper titled "Detection of Alzheimer’s and Parkinson’s Diseases Using Deep Learning-Based Various Transformers Models" (<https://doi.org/10.3390/engproc2024073004>) has been studied rigorously to establish context and strengthen the foundation of this study. Also, it has been added to the reference section.

In the paper page 20 in the References section, we add the following references:

30. Guven M. Detection of Alzheimer’s and Parkinson’s Diseases Using Deep Learning-Based Various Transformers Models. Eng. Proc. 2024;73(1):4. Presented at the 4th International Electronic Conference on Biosensors, 20–22 May 2024. Available online: https://sciforum.net/event/IECB2024. <https://doi.org/10.3390/engproc2024073004>.

**In the paper in the Literature Review Section, page 4, line 151, we add the following paragraph:**

“Recent work by [30] applied transformer-based models, including Swin Transformer, Vision Transformer (ViT), and Bidirectional Encoder Representation from Image Transformers (BEiT), to classify Alzheimer’s and Parkinson’s diseases using brain imaging data. The study utilized a balanced dataset of 450 brain images, achieving classification accuracy exceeding 80%, with ViT demonstrating the highest performance (94.4% accuracy, 94.7% precision). While the results highlight the efficacy of transformer architectures in disease detection, the study has notable shortcomings. The dataset size (450 images) is relatively small, which may limit the generalizability of the findings. Also, the accuracy is not satisfactory in terms of AD disease diagnosis.”

**Reviewer#4 Comments:**

Your manuscript offers a valuable contribution to AI-driven Alzheimer’s disease diagnosis, demonstrating high accuracy and integrating explainability via LIME. The combination of transformers for MRI and RNA sequencing analysis is innovative and well-justified. The literature review is comprehensive, and the methodological framework is presented. However, the manuscript could benefit from additional validation on external datasets, a more detailed discussion on clinical applicability, and minor refinements in writing and formatting. These improvements would enhance the paper’s clarity and real-world impact:

**Comment-1:**

The introduction clearly states the importance of early AD diagnosis and the role of AI in medical imaging, but the research gap could be more explicitly defined to emphasize why transformers are superior to CNNs or traditional machine learning models. Additionally, the claim that "there is no cure for AD" should be softened, as some experimental treatments exist. A brief outline of the paper structure at the end of the introduction would improve readability and guide the reader.

**Response-1:**

**Thank you for your valuable suggestions.**

We have revised the introduction to explicitly define the research gap, emphasizing the advantages of transformers over CNNs and traditional machine learning models. The statement "there is no cure for AD" is replaced as “Currently there is almost no cure for AD except very few experimental treatments.”

**The following paragraph is added in the Introduction section, page 2, 32 lines:**

“A notable advancement in DL is the development of the Transformer architecture, which employs attention mechanisms to capture relationships between elements in data. Initially designed for Natural Language Processing (NLP), the Transformer’s architecture has been adapted for image analysis, resulting in the Visual Transformer (ViT). ViT is better than conventional CNNs because they capture global dependencies in images through self-attention mechanisms. CNNs particularly focus on local patterns, while ViTs can capture intricate patterns in an image due to their ability to extract long-range dependencies across the entire image rather than just local features like CNNs. Also, ViTs offer more flexibility and scalability, especially with large datasets. This model utilizes parallel processing to enhance speed, capacity, and accuracy in image-based applications. Despite these advancements, the application of transformer-based models in AD diagnosis remains relatively underexplored. Notably, Convolutional Neural Networks (CNNs) and SpinalNet have demonstrated considerable accuracy in determining different stages of AD [8]. However, there is a gap in incorporating explainable artificial intelligence (XAI) techniques into transformer-based models. This limitation highlights the need for further investigation into the integration of XAI tools to enhance interpretability and reliability in AD diagnostics. XAI can provide detailed explanations for model predictions, aiding clinicians in understanding the reasons behind diagnostic outcomes. This is particularly important in a critical field like AD detection, where transparency and trust are essential for ensuring clinical adoption and improving patient outcomes.”

**The following brief outline of the paper structure has been added at the end of the introduction to improve readability and guide the reader.**

“The paper’s structure is outlined as follows: Section II summarizes previous works. In Section III, we present the datasets, highlighting the interconnection between input attributes. Section IV details the methodology and architecture of the proposed framework. The experimental approach is elucidated in Section V, and Section VI provides a summary of the paper. Section VII concludes with a discussion of future directions for research.”

**Comment-2:**

The methodology section is highly technical and would benefit from a flowchart or diagram illustrating the proposed model architecture. Additionally, key training parameters (batch size, optimizer, number of epochs) should be summarized in a table to improve clarity. The explanation of LIME should be expanded to include how interpretability was validated—was an expert medical review conducted to confirm whether the AI highlights relevant features?

**Response-2:**

The proposed model architecture is added to enhance clarity in Section 4 as **Figure 1**. The key training parameters (batch size, optimizer, number of epochs) are now summarized in a table (**Table 4**). The explanation of LIME is expanded and written in subsection (5.5) titled: "**Interpretation of the Model from a Medical Perspective”** to include validation through expert medical review, ensuring the highlighted features are clinically relevant.

**Comment-3:**

The reported accuracy of 99% raises concerns about overfitting, especially since deep learning models typically struggle with medical imaging data generalization. The study should include external validation using an independent dataset (e.g., ADNI, OASIS) or a detailed description to confirm robustness. Additionally, statistical measures such as confidence intervals or standard deviations should be included to ensure the reliability of the reported results. While the comparative analysis with other models is strong, it could be further improved by incorporating benchmarks such as ResNet or EfficientNet.

**Response-3:**

To address concerns about overfitting, we experiment with external validation using an independent dataset (**ADNI**) in Section 5.6. The class-wise accuracy of the ADNI dataset is presented in Table 7. In addition, we experiment in Section 5.1 with confidence interval (CI) which is derived from the optimum 5-fold cross-validation. Thus, the possibility of overfitting is avoided.

We add the **confidence interval** (CI) statistical measure in Section 5.1, which is derived from the mean accuracy, number of samples, and **standard deviations**.

The performance of ResNet and EfficientNet models is included in **Table 5.**

**Comment-4:**

Figures 6 and 7 require more detailed captions explaining the significance of their findings. The LIME heatmaps should include clinical validation, ideally with references to known AD biomarkers. Additionally, the results section would benefit from a ROC-AUC curve to provide a more comprehensive evaluation of classification performance.

**Response-4:**

We have modified and updated the captions for Figures 6 and 7 to provide more detailed explanations of their findings. The LIME heatmaps include clinical validation with references to known AD biomarkers **in Section 5.5.** We add the ROC-AUC curve to the results section in Table 5 and Table 4 for a more comprehensive evaluation of classification performance.

**Comment-5:**

The discussion should better address dataset biases, such as demographic imbalances and variations in MRI scanning equipment. The interpretability aspect of LIME should be assessed from a clinical perspective—does the AI highlight regions relevant to AD diagnosis? Furthermore, potential regulatory challenges (e.g., FDA approval for AI-based diagnostic tools) should be briefly discussed.

**Response-5:**

We add a **Discussion section as Section 6** to address dataset biases, including demographic imbalances and variations in MRI scanning equipment. **The following lines are included in this section:**

“The findings of this study underscore the transformative potential of advanced deep learning models, such as Vision Transformer (ViT) and MobileNetV2, in enhancing the accuracy of Alzheimer’s disease (AD) diagnosis through MRI image analysis. ViT’s superior performance, achieving an overall accuracy of 98.6% with a tight 95% confidence interval [97.41%, 99.36%], highlights its ability to capture intricate patterns in brain imaging data that traditional methods might overlook. MobileNetV2, while slightly less accurate, offers a lightweight alternative, making it viable for resource-constrained settings, which broadens the practical scope of AI-driven diagnostics. The integration of Explainable AI (XAI) techniques like LIME further elevates these models by providing interpretable insights, such as highlighting the hippocampus and temporal cortex as key regions in AD progression. This interpretability bridges the gap between complex AI outputs and clinical utility, fostering trust among healthcare professionals who rely on actionable explanations. Extending the analysis to RNA-sequencing data with transformer learning revealed complementary insights, suggesting that multi-modal approaches could refine AD prediction beyond imaging alone. However, the study’s reliance on specific datasets raises concerns about generalizability, as demographic imbalances—age, gender, or ethnicity—may skew model performance across diverse populations. Similarly, variations in MRI equipment and protocols across institutions introduce noise, potentially undermining the models’ consistency in real-world scenarios. These limitations echo broader challenges in AI healthcare research, where dataset quality often dictates success more than algorithmic sophistication. The high AUC scores across AD stages (e.g., 1.00 for NonDemented, 0.96 for MildDemented) from ViT suggest robust discrimination, yet they also prompt questions about overfitting to the training data’s characteristics. From a clinical perspective, aligning AI outputs with known AD biomarkers, like hippocampal atrophy, strengthens diagnostic confidence, but validation against larger, standardized cohorts remains critical. The regulatory landscape adds another layer of complexity, as FDA approval demands rigorous, multi-center trials to ensure safety and efficacy beyond academic benchmarks. Encouragingly, the narrow confidence intervals from 5-fold cross-validation affirm the models’ stability, yet future work must address scalability to diverse clinical environments. Integrating multi-center datasets with standardized protocols could mitigate biases and enhance deployment feasibility. Ultimately, this study lays a foundation for AI to revolutionize AD diagnostics, but its success hinges on overcoming technical, ethical, and regulatory hurdles in tandem.”

**The interpretability of LIME is assessed from a clinical perspective by the following lines, confirming that the AI highlights regions relevant to AD diagnosis in sections 5.5 and 5.6.2.**

**Potential regulatory challenges, such as FDA approval for AI-based diagnostic tools, are discussed in the Discussion and Conclusion Sections.**

**Comment-6:**

Some sections contain long, complex sentences that could be restructured for clarity. The use of abbreviations should be consistent (e.g., CNN should be abbreviated after its first mention). Additionally, minor grammatical redundancies, such as "AI-based artificial intelligence," should be corrected for conciseness.

**Response-6:**

We restructure long, complex sentences for clarity and ensure consistent use of abbreviations. Minor redundancies are corrected for conciseness.
