# Supplementary material for: Leveraging transformers and explainable AI for Alzheimer’s disease interpretability
Source: PLoS One. 2025 May 23;20(5):e0322607. doi: 10.1371/journal.pone.0322607 (PMC12101688; doi:10.1371/journal.pone.0322607)
Supplement: Attachment — Submitted filename: Reviewer Comment.docx [file pone.0322607.s004.docx]

**Reviewer Comment:**

We appreciate the submission of this insightful manuscript addressing the critical challenge of Alzheimer’s disease detection using advanced transformers and computer vision techniques. The study ***presents a compelling approach with high diagnostic accuracy and integrates explainable AI (LIME) to enhance model interpretability, which is commendable***!

However, we recommend a minor revision to strengthen the manuscript further. Specifically, we suggest incorporating a reference to the study titled "***Detection of Alzheimer’s and Parkinson’s Diseases Using Deep Learning-Based Various Transformers Models***"

**<** [**https://doi.org/10.3390/engproc2024073004**](https://doi.org/10.3390/engproc2024073004) **>**

This reference aligns well with your work as it discusses the efficacy of transformer-based deep learning models for neurodegenerative disease detection, providing additional context and supporting evidence for the transformative potential of such models. Including this reference will enhance the credibility of your study and position it within the broader research landscape.

Overall, we find your manuscript well-structured and impactful, and we recommend its acceptance with minor revisions as noted above. ***We look forward to your revised submission***.

Best regards,
