# Supplementary material for: Leveraging transformers and explainable AI for Alzheimer’s disease interpretability
Source: PLoS One. 2025 May 23;20(5):e0322607. doi: 10.1371/journal.pone.0322607 (PMC12101688; doi:10.1371/journal.pone.0322607)
Supplement: Attachment — Submitted filename: Reviewer Comments.pdf [file pone.0322607.s001.pdf]

Response to Reviewer's Comments

Dear Reviewer(s),

We sincerely appreciate the constructive feedback provided by the you. This helped us significantly improve the quality of our manuscript. Below, we have addressed each of the comments in detail and outline the corresponding revisions made to the manuscript.

Reviewer 1 Comments and Our Responses

1. **Structuring and Writing Quality of the Introduction**
   - **Comment:** The introduction could be better structured into segments based on relevance. Connections between sentences weaken as it progresses, and insufficient citations are provided. Additionally, the statement regarding the gap in incorporating XAI techniques into transformer-based models lacks explanation or citations.
   - **Response:** We have restructured the introduction into clearly defined segments for better readability and logical flow. We have also enhanced the writing quality by refining sentence transitions and word choices. For the statement about the gap in incorporating XAI techniques into transformer-based models, we have provided a brief explanation. We also made an effort to provide the relevant citations.
2. **Improvements in the Literature Review**
   - **Comment:** Similar improvements are needed in the literature review. While the content is generally acceptable, the writing style should be enhanced. It would be beneficial to ensure relevance between sentences and to review the word choices throughout. For instance, if you spell out 'Convolutional Neural Network' once, you can simply use the abbreviation 'CNN' in subsequent references..
   - **Response:** We have carefully reviewed and refined the writing style in the literature review to improve readability and maintain relevance between sentences. Word choices have been optimized, and abbreviations like "Convolutional Neural Network" (CNN) are now consistently used after being spelled out once.
3. **Validation of Results Section**
   - **Comment:** The results section needs further validation beyond the comparative analysis with MobileNetV2. Additional off-the-shelf network architectures should be included for better validation.
   - **Response:** We have expanded the results section by including comparative analyses with several additional state-of-the-art architectures, such as Swin Transformers and Skip-connected Convolutional Autoencoders in addition to previously provided Vision Transformers and MobileNetV2. This enhanced validation

provides stronger evidence supporting our claims and improves the robustness of our findings.

4. **Quality of Figures 6 and 7**
    - **Comment:** The quality of Figures 6 and 7 can be improved.
    - **Response:** We have improved the quality of Figures 6 and 7 by resizing them and ensuring visual clarity. Labels, legends, and other graphical elements have been adjusted for better comprehensibility.
5. **Inaccurate Writing Format in Section Headings**
    - **Comment:** Check all section headings for formatting inaccuracies.
    - **Response:** We have thoroughly reviewed and corrected all section headings to adhere to the journal's formatting guidelines. Inconsistencies in capitalization, numbering, and alignment have been addressed.

We believe that these revisions have addressed all of the reviewers' comments comprehensively, and we hope the revised manuscript meets the journal's standards for publication.

Thank you for your time and consideration. We look forward to your feedback.

Sincerely,
Authors.
